# Evolutionary dynamics of the chromatophore genome in three photosynthetic *Paulinella* species

**DOI:** 10.1038/s41598-019-38621-8

**Published:** 2019-02-22

**Authors:** Duckhyun Lhee, Ji-San Ha, Sunju Kim, Myung Gil Park, Debashish Bhattacharya, Hwan Su Yoon

**Affiliations:** 1 Department of Biological Sciences, Sungkyunkwan University, Suwon, 16419 Republic of Korea; 2 Department of Oceanography, Pukyong National University, Busan, 48513 Republic of Korea; 3 Department of Oceanography, Chonnam National University, Gwangju, 61186 Republic of Korea; 4 Department of Biochemistry and Microbiology, Rutgers University, New Brunswick, NJ 08901 USA

## Abstract

The thecate amoeba *Paulinella* is a valuable model for understanding plastid organellogenesis because this lineage has independently gained plastids (termed chromatophores) of alpha-cyanobacterial provenance. Plastid primary endosymbiosis in *Paulinella* occurred relatively recently (90–140 million years ago, Mya), whereas the origin of the canonical Archaeplastida plastid occurred >1,500 Mya. Therefore, these two events provide independent perspectives on plastid formation on vastly different timescales. Here we generated the complete chromatophore genome sequence from *P*. *longichromatophora* (979,356 bp, GC-content = 38.8%, 915 predicted genes) and *P*. *micropora* NZ27 (977,190 bp, GC-content = 39.9%, 911 predicted genes) and compared these data to that from existing chromatophore genomes. Our analysis suggests that when a basal split occurred among photosynthetic *Paulinella* species ca. 60 Mya, only 35% of the ancestral orthologous gene families from the cyanobacterial endosymbiont remained in chromatophore DNA. Following major gene losses during the early stages of endosymbiosis, this process slowed down significantly, resulting in a conserved gene content across extant taxa. Chromatophore genes faced relaxed selection when compared to homologs in free-living alpha-cyanobacteria, likely reflecting the homogeneous intracellular environment of the *Paulinella* host. Comparison of nucleotide substitution and insertion/deletion events among different *P*. *micropora* strains demonstrates that increases in AT-content and genome reduction are ongoing and dynamic processes in chromatophore evolution.

## Introduction

The endosymbiotic integration of a cyanobacterium into a eukaryotic host cell gave rise to the common ancestor of the Archaeplastida that subsequently split into the three primary plastid lineages, Glaucophyta, Viridiplantae, and Rhodophyta^1–3^. This primary endosymbiosis is estimated to have occurred >1.5 billion years ago^4,5^. Primary plastid origin is however exceedingly rare, with the only other known case being the relatively recent (i.e., 90–140 Mya^6^) independent origin of this organelle in photosynthetic *Paulinella* species^7,8^. Photosynthetic *Paulinella*, a lineage of thecate filose amoebae is a member of the Cercozoa (Rhizaria)^9,10^, has two sausage-shaped or U-shaped plastids termed “chromatophores”, which were derived from a “*Synechococcus*-like” alpha-cyanobacterium^11,12^.

Three photosynthetic *Paulinella* species have thus far been reported, two that are freshwater, *P*. *chromatophora*^7^ and *P*. *micropora*^13,14^, and one marine, *P*. *longichromatophora*^15^. These lineages are expected to provide invaluable information because comparing the genome of free-living cyanobacterial relatives to that of the endosymbionts offers fundamental insights into organellogenesis. Three chromatophore genome sequences exist, from *P*. *chromatophora* strain CCAC 0185^16^ and two *P*. *micropora* strains FK01 and KR01^14,17^. Compared to the free-living cyanobacterium, *Synechococcus* sp. WH 5701 that is closely related to the donor lineage, the genomes of chromatophores encode relatively more genes with assigned functions than those that lack annotation, suggesting that the latter set may be involved in responses to environmental change that were lost due to the shift to a homogeneous intracellular environment^16^. However, many genes with known and essential functions have also been lost from the chromatophore genome, suggesting that this organelle is dependent on its host for growth and survival. This idea was supported by the inability of isolated chromatophores to divide autonomously^18^ and more recently, by the finding of specific targeting sequences at the N-terminus of large cytosolic proteins to allow their entry into the chromatophore^19^. When comparing the chromatophore genomes of *P*. *chromatophora* CCAC 0185 and *P*. *micropora* FK01, significant conservation in gene order was found with only a handful of minor gene rearrangements^17^. Moreover, inspection of 681 DNA alignments showed that the vast majority had dN/dS ratios ≪1, implying that chromatophore genomes of *P*. *chromatophora* and *P*. *micropora* are strongly constrained by selection.

As described above, the chromatophore genomes of *Paulinella* species have experienced reductive genome evolution. Compared to free-living *Synechococcus* species, the chromatophore genome (ca. 1 Mbp) was reduced one-third in size, the GC-content (39%) fell when to compared to the donor lineages (~57%), and the substitution rate was elevated^13^. Similar processes have been reported in other host-dependent bacteria, leading to a relatively small genome size, high sequence substitution rate, enriched AT-rich base composition, and metabolic simplification^20–23^. These reductive processes can be roughly divided into two phases. At the onset, many genes that are not adaptive to the new environment in the host are inactivated, resulting in the formation of pseudogenes or the complete loss of genomic DNA through large deletion events. Inactivation and recombination events are frequent, giving rise to genomes with abundant pseudogenes and rearrangements. Examples of genomes in this stage are *Mycobacterium leprae*^24,25^, *Bordetella pertussis*^26^, *Serratia symbiotica*^27^, *Polynucleobacter necessaries*^28,29^, *Sitophilus oryzae*^30^, *Richelia intracellularis*^31^, and the *Epithemia turgida* spheroid body^32^. In the more advanced stages of genome degradation, most of the unnecessary information has been completely lost, resulting in highly reduced genomes with a scarcity of pseudogenes. Some of the genes related to recombination and repair processes may have been lost, impacting genome stability. The speed of degradation slows down significantly in the final stages of genome reduction. Examples of endosymbiotic bacterial genomes in this stage are *Buchnera aphidicola*^33–36^, *Blochmannia* spp.^37,38^, *Calyptogena okutanii,* *C*. *magnifica*^39^, and *Candidatus* Atelocyanobacterium thalassa^40^.

Here we sequenced the chromatophore genome from *Paulinella micropora* NZ27 and from *Paulinella longichromatophora*, the latter being the only known marine photosynthetic *Paulinella* species. Available chromatophore genomes were compared to identify reductive genome dynamics in this clade. We also compared these data to the genomes of free-living cyanobacteria and Archaeplastida plastids to better understand the evolutionary history of photosynthetic organelles of primary endosymbiotic origin.

## Results and Discussion

### Chromatophore genome structure in *P*. *longichromatophora* and *P*. *micropora*

**Figure 1:**
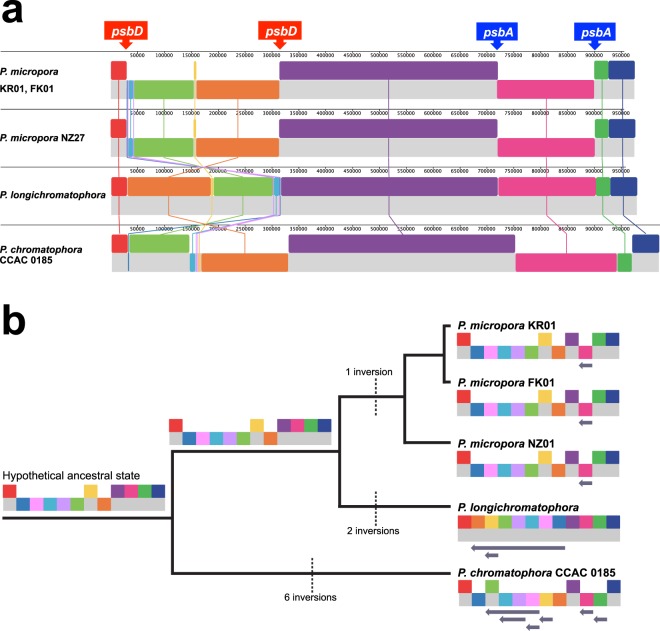
(**a**) Gene synteny comparison of the chromatophore genomes from *Paulinella longichromatophora*, *P*. *micropora* NZ27, *P*. *micropora* KR01, *P*. *micropora* FK01, and *P*. *chromatophora* CCAC 0185. (**b**) Synteny blocks are simplified on the tree and inversion events were calculated using parsimony. Hypothetical ancestral states were constructed by mapping chromatophore genes to the *Synechococcus* sp. WH 5701 genome.

The circular chromatophore genome of *P*. *longichromatophora* was 979,356 bp in length and comprised 867 protein coding sequences (CDS) with six rRNAs and 42 tRNAs. The GC-content of this genome was 38.8% (Table 1). The length, GC-content, and number of CDS in *P*. *longichromatophora* were intermediate to those of *P*. *chromatophora* and *P*. *micropora*. Among these *Paulinella* chromatophore genomes, we found 12 syntenic blocks (Fig. 1a). Synteny comparison showed that there were six inversions between *P*. *longichromatophora* and *P*. *chromatophora* and only three inversions between *P*. *longichromatophora* and *P*. *micropora*. This result is consistent with the multi-gene phylogeny (Supplementary Fig. S1), which showed that *P*. *longichromatophora* is more closely related to *P*. *micropora* than to *P*. *chromatophora*. In the case of *Paulinella micropora* NZ27, the chromatophore genome was 977,190 bp in length, the GC-content was 39.9%, and 863 CDSs were identified. The genome structure was conserved among the three *P*. *micropora* strains. Overall, we find that genome structure of photosynthetic *Paulinella* species is highly constrained.

**Table 1:** A comparison of chromatophore genomes of *Paulinella longichromatophora*, *P. micropora* NZ27, *P. micropora* KR01, *P. micropora* FK01, and *P. chromatophora* CCAC 0185.

	Length (bp)	GC-content (%)	CDS	rRNA	tRNA	Pseudogene	GenBank Accession Number
*P*. *longichromatophora*	9,79,356	38.8	867	6	42	5	MG264610
*P*. *micropora* NZ27	9,77,190	39.9	863	6	42	3	MG976688
*P*. *micropora* KR01	9,76,991	39.9	860	6	42	4	KX897545
*P*. *micropora* FK01	9,77,204	39.9	860	6	42	4	KY124271
*P*. *chromatophora* CCAC 0815	1,021,616	38	878	6	42	11	NC_011087

Between these synteny blocks, some inversion breakpoints were located at the duplicated genes (i.e., *psbA* and *psbD*, respectively). Few repeats are found in the late stages of genome reduction and duplicated genes (e.g., *psbA*, *psbD*) at some inversion breakpoints in chromatophore genomes appear to mediate inversions due to intra-molecular recombination^14,41,42^. In addition, using the *Synechococcus* sp. WH 5701 genome to infer the common ancestral state, we calculated, using parsimony, the number of chromatophore inversion events (Fig. 1b). *P*. *micropora* contained one inversion, *P*. *longichromatophora* had two inversions, whereas *P*. *chromatophora* had six inversion events when compared to the putative ancestral state.

### Genomic changes from cyanobacteria to chromatophores

**Figure 2:**
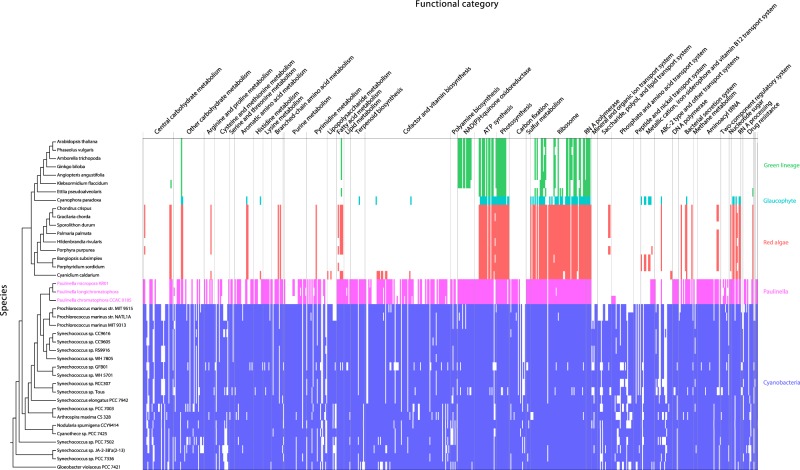
Heatmap with genomes of chromatophores, cyanobacteria, and Archaeplastida plastids categorized by KEGG pathways annotated as Orthologous Gene Families (OGFs). A total of 401 OGFs (x-axis) are ordered according to their KEGG annotation category. Each tick indicates genes present in the OGFs; red indicates red algae, green are green lineage species, cyan indicates glaucophytes, pink indicates *Paulinella*, and blue indicates cyanobacteria. On the left side of heatmap, the tree was constructed in a concatenated phylogenomic analysis of amino acids data using 20 orthologous genes from a total of 40 representative genomes in Archaeplastida plastids, cyanobacteria, and chromatophore genomes of *Paulinella* lineages (Supplementary Table S6).

We compared chromatophore genomes with cyanobacterial and other plastid genomes. The clustering of proteins from three chromatophore genomes, 17 Archaeplastida plastid genomes, and 20 cyanobacterial genomes yielded 3,348 Orthologous Gene Families (OGF) that are shared by at least five taxa. Among these, 584 OGFs, with some additional orthologs were examined in detail (Fig. 2, Supplementary Fig. S2). The plastids of the green lineage (Viridiplantae), red algae, and glaucophytes lost most OGFs, with the exception of photosynthesis and ribosome-related genes. Red algal plastids contained more OGFs in other metabolic pathways when compared to its two sister taxa. In the evolutionary history of Archaeplastida, red algae and the green lineage underwent differential gene loss after their split from a single common ancestor^43^. Moreover, endosymbiotic gene transfer (EGT) has played an important role in the establishment of primary plastids^44,45^, whereby a total of 956 plastid-derived genes have been relocated to the nuclear genomes of green plants (886 genes), glaucophytes (48 genes), and red algae (22 genes)^43^.

**Figure 3:**
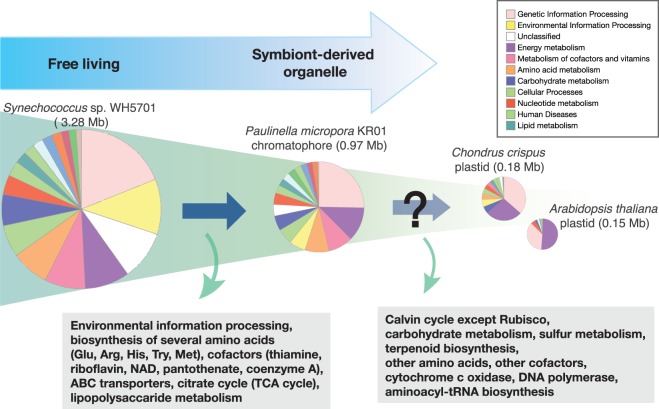
Schematic figure showing the process of organellogenesis in *Paulinella*, starting from a free-living cyanobacterium to the permanent chromatophore. Lost KEGG pathways from cyanobacteria to chromatophore, and different pathways between chromatophore and Archaeplastida plastids are listed.

As reported in a previous study^16^, genome reduction has progressed to a smaller extent in the chromatophore genome of *Paulinella* when compared to Archaeplastida plastids (Fig. 3, Supplementary Table S1). In particular, the citric acid cycle (TCA), environmental information processing, and ABC transporters genes underwent loss. Several amino acid biosynthetic pathways (Glu, Arg, His, Try, Met), cofactors (thiamine, riboflavin, NAD, pantothenate, coenzyme A), and lipopolysaccharides metabolism were also completely lost. In addition, some pathways of amino acid biosynthesis (Gln, Asp, Asn, Lys, Cys, Thr, Tyr, Phe), lipid metabolism, biotin biosynthesis, and the Sec (secretion) system were partially deleted. In contrast, photosynthesis, genetic information processing, and fatty acid biosynthesis functions were retained in chromatophores, as in the plastids of Archaeplastida.

There are two possible routes for organelle genome reduction, outright gene loss or relocation to the nuclear genome via EGT. Lipopolysaccharide synthesis genes may provide an example of complete loss because these were presumably no longer needed in the homogeneous intracellular environment. Lipopolysaccharides are large molecules present in the outer membrane of Gram-negative bacteria^46^. The original outer membrane of the chromatophore is proposed to have been lost^8^. Some of the lost or incomplete pathways in chromatophores were compensated for by nuclear genes in the case of *P*. *chromatophora* CCAC 0185^47^. For example, missing components of the Met, Ser, Gly, Arg, His, aromatic amino acid, and purine biosynthesis pathways were filled by proteins encoded on the nuclear genome. Interestingly, these nuclear genes were primarily not derived from chromatophore DNA. At least 229 nuclear genes were acquired via horizontal gene transfer (HGT) from various bacteria, and 25% putatively arose through EGT from the chromatophore genome^47^. In a more recent analysis of the chromatophore proteome, Singer *et al*.^19^ found that the large majority of imported proteins are host (i.e., nuclear gene)-derived or of unknown origin, suggesting that a major redeployment of *Paulinella* genetic resources was the primary means of domesticating the organelle, in addition to HGT and EGT.

Even though lost genes in the endosymbiont can be counteracted by the host, some interesting trends are apparent within the retained inventory. In the case of the obligate endosymbiont *Polynucleobacter*, genes related to DNA, RNA and protein metabolism or energy production were lost less frequently than transporters or genes involved in growth, sensing or regulation^29^. The chromatophore genome of *Paulinella* also shows this pattern. Genome comparisons among primary endosymbionts (i.e., UCYN-A1, the *Epithemia turgida* spheroid body and the chromatophore of *P*. *chromatophora*) showed that 62% of chromatophore encoded genes was shared with both of these endosymbionts^48^, suggesting that some core genes are retained despite independent evolution.

Pairwise comparisons of the ratio of substitution rates at non-synonymous and synonymous sites (dN/dS) in free-living *Synechococcus* showed that genes remaining in the chromatophore have lower dN/dS values. *Synechococcus* genes were grouped into three different categories (Supplementary Fig. S3, Table S2): genes retained in all chromatophores (GROUP-1), genes present in the common ancestor of all chromatophores but lost in some lineages (GROUP-2), and genes lost during the transition to chromatophore (GROUP-3). A significant increase in the average value of dN/dS from GROUP-1 (0.405) through GROUP-2 (0.508) to GROUP-3 (0.567) was observed (t-test, *p*-value < 0.05), suggesting that retained genes were under stronger purifying selection. The average Codon Adaptation Index (CAI) values of *Synechococcus* species were significantly greater in GROUP-1 (0.754) than GROUP-3 (0.732) (t-test, *p*-value < 0.001), implying that the low CAI genes were more readily lost than high CAI genes. Adaptive codon bias is correlated with gene expression level^49–51^, thus *Synechococcus* genes remaining in the chromatophore may have higher expression. This finding of gene loss reflecting lower CAI has also been reported in *Buchnera* strains that are insect endosymbiotic bacteria^52^. Although dN/dS and CAI values of free-living *Synechococcus* species do not represent directly the evolutionary dynamics of intracellular chromatophores, we assume this result conveys useful insights into the evolutionary trajectory of chromatophore gene loss. That is, the impact of selection and codon usage bias in ancestral free-living cyanobacteria are broadly applicable to (and conserved) in captured endosymbiont genomes.

In order to elucidate the dynamics of mutation and selection in chromatophore genes, the dN/dS ratio was calculated using data from free-living *Synechococcus* strains and chromatophores. It turned out however that the synonymous sites were largely saturated, thereby weakening this analysis. To address this issue, the RELAX program^53^ was used to estimate the relaxation or intensification of selection in chromatophore genes when compared to free-living alpha-cyanobacteria (Supplementary Table S3). Among 542 OGF that were well conserved among all species, 325 returned significant results. Among these, only 22 OGFs had intensified selection, whereas the remainder (303 OGFs) showed relaxed selection in chromatophore genes. This category encompassed a variety of functional categories, implying that relaxed selection is occurring at the genome level. This result is consistent with analysis of bacterial endosymbionts in insect species^53^.

**Figure 4:**
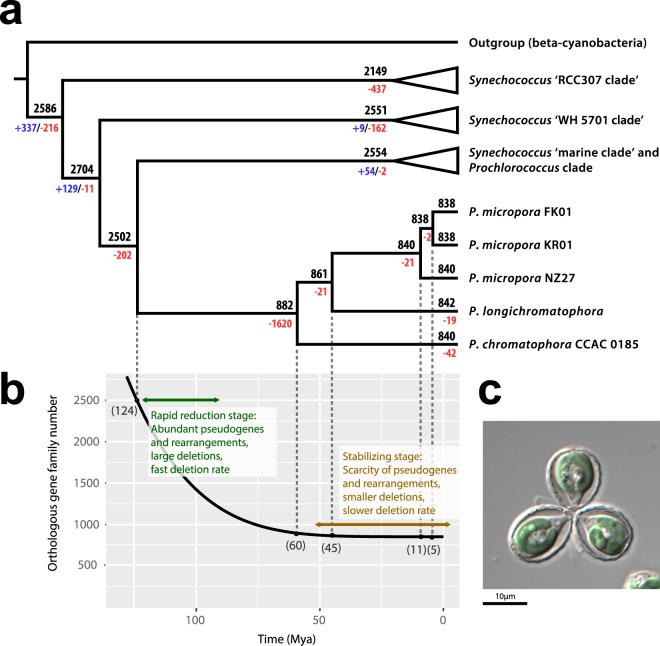
(**a**) Summary of orthologous gene families (OGF) loss events across the phylogenetic tree of photosynthetic *Paulinella*. This figure presents the total number of OGFs in each species or node estimated by Dollo parsimony. The numbers on branches of the phylogenetic tree correspond to expanded (blue), contracted (red), or inferred ancestral (black) OGFs along each lineage. (**b**) Proposed evolutionary model of OGFs in the chromatophore genome of *Paulinella*. The split time was estimated according to SSU rRNA lognormal molecular clock calculations. Numbers inside the parentheses indicate the estimated split time (Mya). (**c**) Light microscopy image of *Paulinella micropora* KR01.

The transition to a host-dependent lifestyle is often coupled with changes in how evolution proceeds^23,54,55^. Intracellular environments cause relaxed selection on many metabolic functions that are no longer required (or are detrimental [e.g., a competing TCA cycle in the chromatophore]) in a host cellular environment. Population size is also critical because reduction in population size lowers the efficacy of selection, favoring a greater role for random genetic drift. In the case of obligate endosymbionts, restriction to hosts and bottlenecks at the stage of inoculation of new hosts will enhance this process due to Muller’s ratchet^56–59^. Previous studies^60,61^ have shown however that although separate subpopulations will accumulate deleterious mutations rapidly, the symbiont population as a whole is protected from extinction due to strong selection for host survival. In the case of *Paulinella*, population expansion is integrated because the chromatophores divide synchronously with the host^62^. Regardless of the details of this complex host-organelle interaction, what is clear is that Muller’s ratchet does not lead to genome extinction in chromatophores (as also found in other plastids) because the genome size has stabilized (see below, Fig. 4b) and many of the retained genes remain essential for host survival. The observation of relaxed selection on chromatophore genes when compared to free-living alpha-cyanobacteria may, therefore, be explained by the homogeneous intracellular environment. This aspect is balanced by very strong selection for the retention of a core set of genes that are critical for organelle function (photosynthesis). If these processes play out in an expanding population, then rare beneficial mutations (including EGT or HGT), may rise to dominance (i.e., through a selective sweep) despite the intracellular, asexual environment that the organelles reside in.

### Gene content differences among chromatophore genomes

Because of the presence of duplicated genes, gene content was compared using orthologous gene families (OGFs) rather than gene numbers. More than 90% of the genes (799 OGFs) were shared between the chromatophore genomes of *Paulinella* species. Some of the differentially lost genes were assigned to specific pathways, as found in the case of the *ruvA/ruvB/ruvC* genes that were lost only in *P*. *longichromatophora*. The chromatophore genome of *P*. *longichromatophora* shared more OGFs with *P*. *micropora* (834) than with *P*. *chromatophora* (819), supporting the sister group relationship of these former species (Supplementary Fig. S1). The novel genome from *P*. *micropora* NZ27 strain had only two different genes (i.e., oxidoreductase and hypothetical protein) when compared to other *P*. *micropora* strains (KR01 and FK01). Unique genes in chromatophore genome of each species were less than 3% (~21 OGFs) of total genes and showed no specific functional trends in KEGG pathway analysis (Supplementary Table S4).

To investigate the history of chromatophore genome reduction, we constructed OGFs from chromatophore proteins and from phylogenetically closely related *Synechococcus*/*Prochlorococcus* species. Ancestral OGF numbers with gain and loss were estimated (Fig. 4a). In addition, divergence times of photosynthetic *Paulinella* species were estimated using a lognormal relaxed clock with 18S rRNA (Supplementary Fig. S4). A previous estimate for the origin of *P*. *chromatophora* was 90–140 Mya^6^. Our estimate (124 Mya), after adding three additional photosynthetic *Paulinella*, was consistent with the previous finding. The estimate of the ancestral OGF number and the molecular clock analysis were used to derive a model of gene family reduction (Fig. 4b). After a former free-living cyanobacterium became the endosymbiont (124 Mya), genes were lost very rapidly during the early stages of plastid evolution. When photosynthetic *Paulinella* species diverged from a common ancestor (60 Mya), 1,620 OGFs were lost, leaving 882 OGFs in the chromatophore; i.e., 65% of the gene inventory in the ancestral free-living cyanobacterium was lost outright. Subsequently, gene loss slowed down and only 40~44 OGFs were lost during the past ca. 60 Mya. Therefore, a small proportion (<5%) of coding sequences was differentially lost and rearrangement occurred rarely among these three species. It should be noted that the molecular analysis usually provides a minimal age for the divergence, hence our estimated divergence times are the minimal ages, and actual divergence time could be much older. Moreover, there is a possibility that extinct alpha-cyanobacteria are the closest relatives of chromatophores. Nonetheless, the current genome reduction model for the chromatophore using available data suggests that the rate of genome reduction has slowed down and genome size has stabilized after diversification of *Paulinella* species. In addition to this result, a high proportion of conserved coding regions is found among species and there is a scarcity of pseudogenes, suggesting that chromatophores in *Paulinella* are in the “stabilizing stage” of endosymbiont genome reduction (see below, Fig. 4).

When comparing genomes of endosymbiont lineages, rearrangement and gene content comparisons should be considered in the context of their divergence time. In the early stages of genome reduction, lost genes can vary conspicuously among all categories and considerable rearrangement may occur among species. When endosymbionts are in the “stabilizing stage” of genome reduction, fewer rearrangements will occur and most genes will be conserved among species. It should be kept in mind that common genes lost among species could have been purged before divergence or independently lost after the species split from each other. Most of the losses in the chromatophore genome of *Paulinella* were likely to have occurred before the basal split of photosynthetic species. Nevertheless, independent loss after speciation also happened as in the case of oxidoreductase (see below, Supplementary Fig. S5).

### Genomic difference among *Paulinella micropora* strains

We searched for recently derived genomic differences between *P*. *micropora* strains (KR01 isolated from Korea, FK01 isolated from Japan, and NZ27 isolated from New Zealand). There was no difference among these strains with regard to gene content, except for two additional genes being present in NZ27, which have been pseudogenized in KR01 and FK01 (Supplementary Table S4). One of these pseudogenized genes is an oxidoreductase that provides an excellent example of progressive gene loss (Supplementary Fig. S5). This gene is intact in NZ27 and in *P*. *longichromatophora*, but has been pseudogenized in the other three *Paulinella* lineages and is at different stages of loss: i.e., there are stop codons at position 673 due to a single substitution (CAA to TAA) in both KR01 and FK01. Additionally, there is a large deletion (181 bp) only in KR01, suggesting future complete loss. In *P*. *chromatophora* CCAC 0185, it was challenging to uncover the history of this gene: i.e., two flanking conserved hypothetical proteins are conserved in all *Paulinella*, but this intergenic space was difficult to align with other oxidoreductases, suggesting complete gene loss. It is interesting that oxidoreductase gene inactivation resulted from two independent events in the ancestor of KR01 and FK01, and *P*. *chromatophora* CCAC 0185. The ortholog of this oxidoreductase was pyridoxal reductase involved in vitamin B6 metabolism^63,64^. Furthermore, one-half of the genes involved in this pathway have been shed in the chromatophore, suggesting that this gene is no longer under selective constraints leading to its loss.

Nucleotide substitution and insertion/deletion events were analyzed between *P*. *micropora* NZ27 and KR01 strains. A total of 22,190 positions (22.7 SNPs/kb) showed single nucleotide polymorphisms (SNPs). These SNPs were more concentrated in noncoding regions (35.9 SNPs/kb) than in coding regions (19.7 SNPs/kb). We also detected a total of 211 insertion or deletion events (indels): 138 single-base indels, 55 indels of 2 to 15 bases, 14 indels of 16 to 42 bases, and 4 large deletions (97 to 136 bases). Compared to SNPs, indels were also more common in noncoding regions (1.05 indels/kb) than in coding regions (0.0263 indels/kb), demonstrating that frameshifts caused by indels could lead to loss of function in proteins. Moreover, the distribution of indels was analyzed among mononucleotide runs (“homopolymers”) (Supplementary Table S5) and found to be more frequent in longer homopolymers.

To determine what types of changes accrued when compared to the ancestral chromatophore, SNPs and indels between KR01 and FK01 were compared to NZ27 and the ancestral state was reconstructed using parsimony (Supplementary Fig. S6). As expected, transitions were more frequent than transversions. However, even among transitions, GC to AT changes were significantly greater than AT to GC (Chi-square test, *p*-value < 0.001). Using these directional base changes, we calculated the relative per-site mutation bias that quantifies the underlying mutational biases^23^. If the genome is under mutational equilibrium, this relative per-site mutational bias (33%) should be equal to the current GC-content (39.9%). However, this was not the case for chromatophore genomes, suggesting that selection on base composition could play a role in the process. This result suggests that AT-enrichment is an ongoing process in chromatophore genomes. This change in GC-content can be explained by the universally biased mutation towards AT in bacteria^65^ and continued relaxed selection on endosymbiont DNA. In the case of indels, insertion events (26) outnumbered deletion events (8), however, the total change was −224 bp because of large deletions, suggesting that genome reduction is also ongoing.

Different mechanisms cause genome reduction in endosymbionts. Insertion of sequences and other mobile DNA can act as molecular mechanisms that contribute to early gene inactivation and deletion in endosymbionts^23^. Moran *et al*.^66^ provided a model of stepwise symbiont genome erosion in *Buchnera aphidicola*. The shift toward high AT-content leads to an increased occurrence of A/T homopolymers, and replication slippage occurs, which results in small indels. These indels lead to frameshifts and gene inactivation, and eventually loss of DNA via large deletions. The chromatophore genome also shows a shift toward high AT-content and the frequency of indels was higher in longer homopolymers, which were mostly A or T. However, this was not as pronounced as in the *Buchnera aphidicola* case, implying that other mechanisms might play bigger role in chromatophore genomes. As in the case of oxidoreductase in photosynthetic *Paulinella*, indels and SNPs without homopolyers can also cause inactivation of genes. After inactivation, DNA removal occurs because mutational bias favors deletions over insertions in bacterial genomes^67^.

## Conclusion

Our genome reduction model demonstrates that chromatophore genomes in *Paulinella* species are in the “stabilizing stage” of DNA loss (Fig. 4). Even though this process has slowed down, many chromatophore genes remain under relaxed selection when compared to homologs in free-living cyanobacterial relatives. Moreover, chromatophore genomes are much larger than Archaeplastida plastids (see Fig. 3). Will chromatophore genome size eventually reduce to that of plastids? We have no way of predicting so far into the future even though the major “rules” of permanent photosynthetic endosymbiont evolution seem to be conserved among the different lineages. The focus of comparative studies should now turn to the nuclear genome of *Paulinella* species to elucidate their role in organelle formation.

## Materials and Methods

### Sampling, isolation, DNA extraction, genome amplification

*Paulinella longichromatophora* was collected from Gomso Bay on the western coast of Korea (35° 31′11.40″N, 126° 29′0.65″E) on March 8, 2015. Using a capillary pipet, each cell was individually isolated and washed several times with sterile DY-V medium. For shotgun genome sequencing, DNA from ~3–15 isolated cells were amplified using the REPLI-g Mini Kit (Qiagen, Santa Clarita, California, USA) and MALBAC kit (Yikon Genomics, Taizhou, Jiangsu, China).

*Paulinella micropora* strain NZ27 was collected from Nga Manu Nature Reserve, Waikanae, Wellington, New Zealand (40°51′39.96″S 175°03′36.63″E) on April 4, 2009. Cells were isolated into culture and maintained in the DY-V medium at room temperature in flat plastic culture flasks. The DNeasy Plant Mini Kit (Qiagen) was used for DNA extraction.

### Chromatophore genome sequencing, assembly, and annotation

In the case of *Paulinella longichromatophora*, next-generation sequencing with the Ion Torrent PGM platform (Thermo Fisher Scientific, Santa Clarita, California, USA) was used to generate data. A sequencing library (400 bp size selection) was constructed using the Ion Xpress Plus gDNA Fragment Library Kit, and genome sequencing was conducted with the Ion PGM Template OT2 400 Kit and the Ion PGM Sequencing 400 Kit following manuals provided by Thermo Fisher Scientific. *Paulinella micropora* strain NZ27 was sequenced using a Hiseq2500 platform (Illumina) at a commercial sequencing company (DNA Link Inc., Seoul, Korea). A sequencing library was constructed using the Truseq DNA PCR-Free kit (Insert 550 bp). The chromatophore genome was assembled using CLC Genomics Workbench 5.5.1 (CLC bio, Aarhus, Denmark) and a MIRA assembler that was incorporated in Ion Server (Thermo Fisher Scientific). Genes were identified using the BLASTx algorithm^68^ by comparing the predicted ORFs to the NCBI GenBank database. Gene annotation was conducted using Geneious 6.1.6 (http://www.geneious.com/), RNAmmer 1.2 Server^69^, and tRNAscan-SE 1.21^70^. For gap filling, polymerase chain reaction (PCR) amplification was done with an initial denaturation step at 95 °C for 10 min, followed by 35 cycles of 95 °C for 1 min, 50–55 °C for 1 min, and 72 °C for 1 min, concluding with a 10 min extension at 72 °C. Amplified products were purified by QIAquick PCR Purification Kit (Qiagen) and QIAquick Gel Extraction Kit (Qiagen), and sent to Macrogen Inc. (Seoul, Korea) for sequencing.

### Phylogenomic analysis

A total of 35 representative genomes from marine, euryhaline, and freshwater *Synechococcus* and *Prochlorococcus* species were included with the five photosynthetic *Paulinella* strains for the phylogenetic analysis based on concatenated amino acids sequence data (Supplementary Table S6). *Gloeobacter violaceus* PCC 7421 was used as the outgroup. The orthologous genes were clustered using OrthoFinder^71^ and alignment was done using MAFFT v7.305b^72^. A total of 406 conserved genes were used to reconstruct the phylogeny. The maximum likelihood phylogeny was reconstructed with IQ-TREE version 1.4.4^73^ using the evolutionary model selected with the IQ-TREE build-in model selection function. For the concatenated tree reconstruction, an independent model for each partition of the concatenated data was used. Support values were estimated using 1,000 replicates of the ultrafast bootstrap implemented in IQ-TREE^74^. For the tree shown in Fig. 2, the same method was used using 20 orthologous genes concatenated amino acids alignment from a total of 40 representative genomes in Archaeplastida plastids, cyanobacteria, and chromatophore genomes of *Paulinella* lineages (Supplementary Table S6).

### Genome structure comparison

Synteny comparison was done using the progressive Mauve alignment algorithm in Geneious 6.1.6 (http://www.geneious.com/). Hypothetical ancestral states were constructed by mapping chromatophore genes to the *Synechococcus* sp. WH 5701 genome. Despite the presence of massive rearrangements, genes flanking the break points were identified and assumed to represent the ancestral state. Genome rearrangement events were counted by UniMoG: a unifying framework for genomic distance calculation and sorting based on DCJ^75^.

### Orthologous Group Families (OGF) analysis

The Orthologous Group Families (OGF) of proteins in cyanobacteria, plastids from Archaeplastida, and chromatophores were clustered using OrthoFinder^71^ (Supplementary Table S6). KEGG pathways^76^ were annotated using BlastKOALA^77^ and a heatmap was constructed using a Python script and Microsoft Excel (Microsoft Corporation, Redmond, Washington, USA). Ancestral OGF numbers with gain and loss were estimated using the Dollo parsimony principle^78^.

### Estimation of substitution rates at non-synonymous and synonymous sites (dN/dS) and codon adaptation index

For calculating the ratio of substitution rates at non-synonymous and synonymous sites (dN/dS), each ortholog was aligned with Clustal Omega^79^ and then re-aligned with PAL2NAL^80^. Maximum likelihood estimates of dS and dN were computed using codeml from the PAML package (runmode −2)^81^. To avoid the problem of insufficient sequence divergence or saturation^82^, genes with dS values >2 and <0.01 were discarded. The dN/dS value was calculated among three free-living *Synechococcus* sp. (CC9616, RS9916, WH 7805) and averaged dN/dS values were used. Codon Adaptation Index (CAI) values of *Synechococcus* sp. WH 5701 were calculated using CAIcal^83^. The codon usage table of *Synechococcus* sp. CC9311 was used to calculate the CAI value. For detecting relaxed selection, a general hypothesis testing framework (RELAX)^53^ on the webserver Datamonkey^84^ was used. Given two subsets of branches in a phylogeny, RELAX can determine whether selective strength was relaxed or intensified in one of these subsets relative to the other.

### Molecular clock analysis

We added the 18S rDNA sequences of *P*. *longichromatophora*, *P*. *micropora* NZ27, and KR01 to the xml data file provided by Delaye *et al*.^6^. Small subunit rRNA sequences from Rhizaria and Stramenopila from the SILVA database were used. Sequences were aligned with MUSCLE^85^. BEAUTi was used to revise the original xml file. Time trees were inferred by using a lognormal relaxed molecular clock as implemented in BEAST 2^86^. We followed the previous work^6^ priors and calibration scheme: a) the origin of rhizosolenid diatoms, which is known with high confidence (91.5 ± 1.5 Mya); b) a minimal time divergence of pennate diatoms (80 Mya); c) a minimal time divergence for diatoms (133.9 Mya); d) a minimal time divergence of Euglyphidae 40 Mya; and e.1) a time estimation of the divergence of Rhizaria from Stramenopila ~1232 Mya.

### Nucleotide substitution and insertion or deletion events

Nucleotide substitution, insertion/deletion events, and homopolymers were analyzed using Geneious 6.1.6 (http://www.geneious.com/) and customized Python scripts. To reconstruct the ancestral state of *P*. *micropora* KR01 and FK01, the chromatophore genomes of *P*. *micropora* KR01, FK01, and NZ27 strains were aligned, and single nucleotide substitution and insertion/deletion events between KR01 and FK01 were searched in NZ27. Sequence shared with NZ27 was considered as the ancestral state of KR01 and FK01. To minimize some bias in the substitution changes, we calculated the number of directional nucleotide change in each region and divided each value by the frequency of the original nucleotide^66^. After obtaining the relative frequency of each class of nucleotide substitution, we transformed these values to make the total sum of the relative frequencies equal to 100%. Finally, we grouped the changes that could not be differentiated because the mutated and complementary strands could not be distinguished.

## Supplementary information

Supplementary Figures S1–6

Table S1

Table S2

Table S3

Table S4

Table S5

Table S6

## Data Availability

Chromatophore genomes of Paulinella longichromatophora (MG264610) and Paulinella micropora NZ27 (MG976688) are deposited at the NCBI archive. The phylogenetic data supporting this article have been uploaded as part of the Supplementary Material.
