# Supplementary material for: Evolutionary dynamics of the chromatophore genome in three photosynthetic Paulinella species
Source: Sci Rep. 2019 Feb 22;9:2560. doi: 10.1038/s41598-019-38621-8 (PMC6384880; doi:10.1038/s41598-019-38621-8)
Supplement: Supplementary file 1 — Supplementary Figures S1–6 [file 41598_2019_38621_MOESM1_ESM.pdf]

SUPPLEMENTARY INFORMATION

Evolutionary dynamics of the chromatophore genome in three photosynthetic *Paulinella* species

Duckhyun Lhee[a], Ji-San Ha[a], Sunju Kim[b], Myung Gil Park[c],

Debashish Bhattacharya[d], Hwan Su Yoon[a, 1]

Supplementary Tables

Table S1. Detailed functional comparison among chromatophores (*Paulinella chromatophora* CCAC 0185, *Paulinella micropora* KR01), cyanobacteria (*Synechococcus* sp. WH 5701, *Synechococcus* sp. CC9605), and Archaeplastida plastids (*Chondrus crispus*, and *Arabidopsis thaliana*). Green box indicate KEGG modules that most of genes were conserved compared to that of *Synechococcus* species. Yellow box indicate KEGG modules that some of genes were remained compared to that of *Synechococcus* species. Red box indicate KEGG modules that no genes were found compared to that of *Synechococcus* species.

Table S2. Ratio of substitution rates at non-synonymous and synonymous sites (dN/dS) and Codon Adaptation Index (CAI) values of *Synechococcus* genes that were grouped into three different categories: genes retained in all chromatophores (GROUP-1), genes present in the common ancestor of all chromatophores but lost in some lineages (GROUP-2), and genes lost during the transition to chromatophore (GROUP-3).

Table S3. RELAX results of chromatophore and alpha cyanobacteria orthologous gene sets. The selection intensity parameter (k) describes the relationship of dN/dS in the test (chromatophore) and the reference (cyanobacteria) branches. Selection on test branches is intensified (k >1) or relaxed (k < 1) compared with background branches. A likelihood ratio test (LRT) was used to test whether k significantly deviated from one.

Table S4. List of orthologous gene families that differentially lost in chromatophore genomes of photosynthetic *Paulinella* lineages. Genes were classified following KEGG pathway.

Table S5. Number and frequency of indels between *Paulinella micropora* NZ27 and KR01 strain for different length of homopolymer.

Table S6. List of cyanobacteria, Archaeplastida plastids, and photosynthetic *Paulinella* species used in this study.

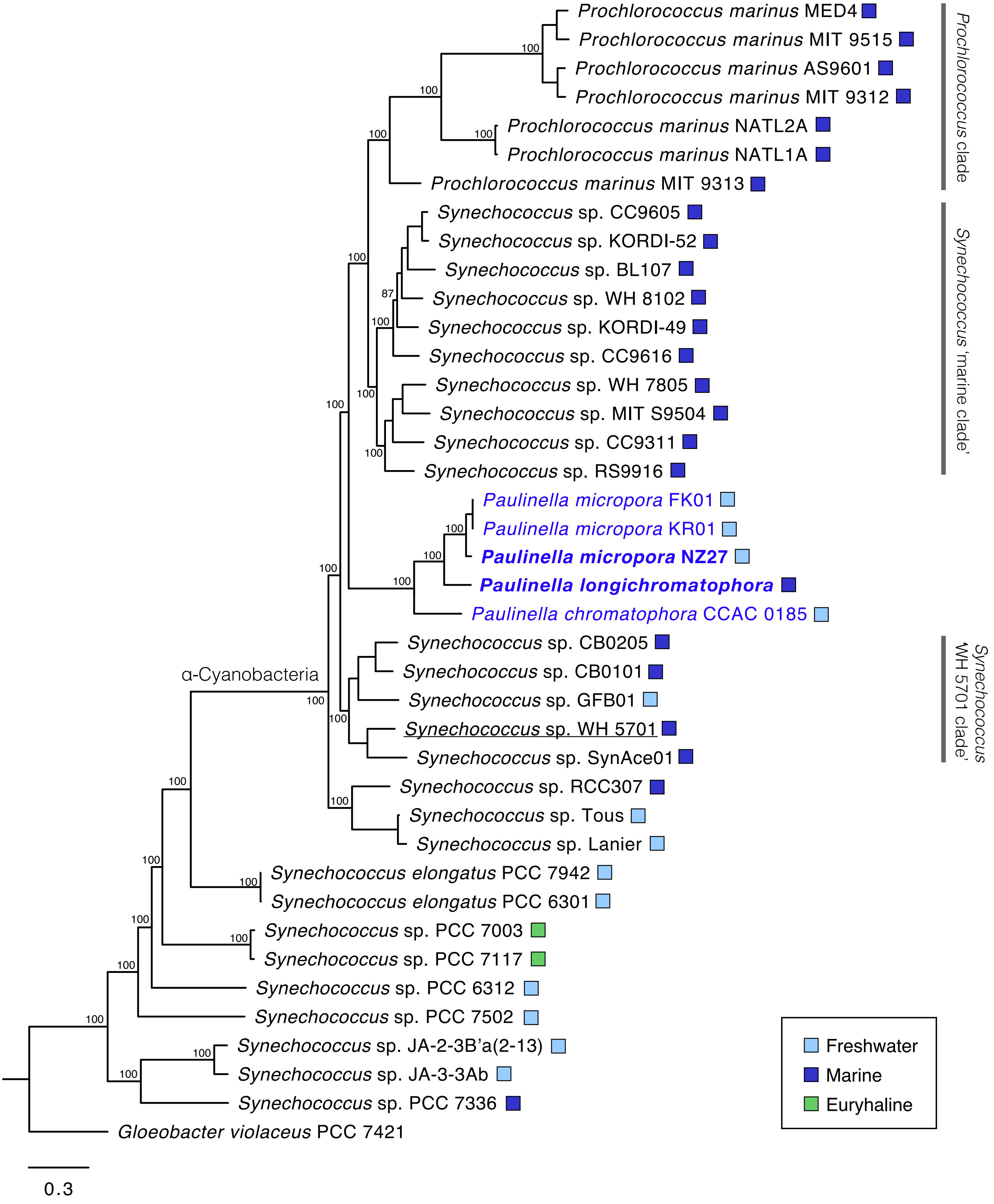

Figure S1. Phylogenomics of cyanobacteria and photosynthetic *Paulinella*. A total of 406 conserved genes were used to generate a maximum-likelihood phylogenomic tree. *Gloeobacter violaceus* PCC 7421 were used as the outgroup.

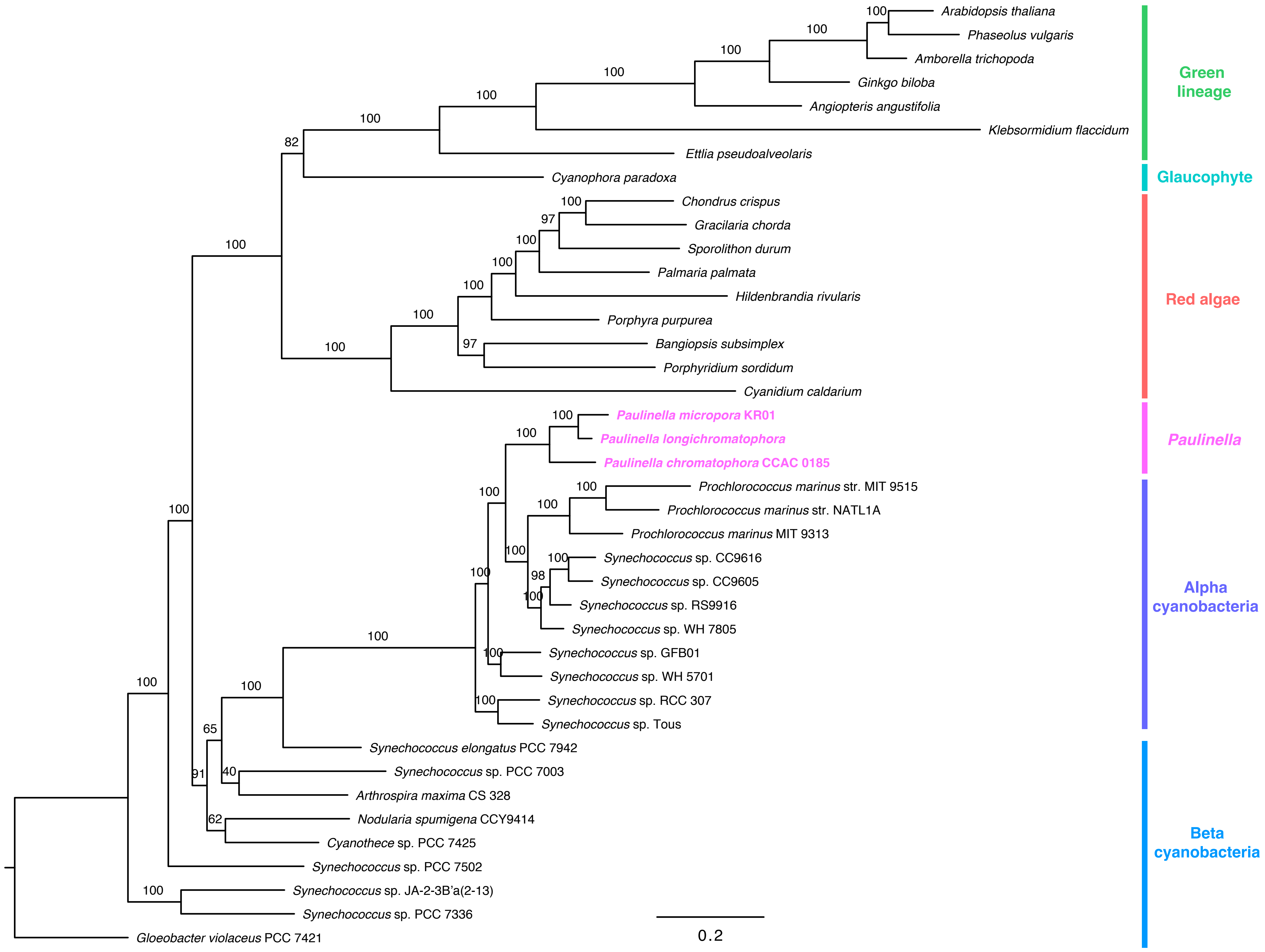

Figure S2. Phylogenomics of Archaeplastida plastids, cyanobacteria, and chromatophore genomes of *Paulinella* lineages. A total of 20 conserved genes were used to generate a maximum-likelihood phylogenomic tree. Gloeobacter violaceus PCC 7421 were used as the outgroup.

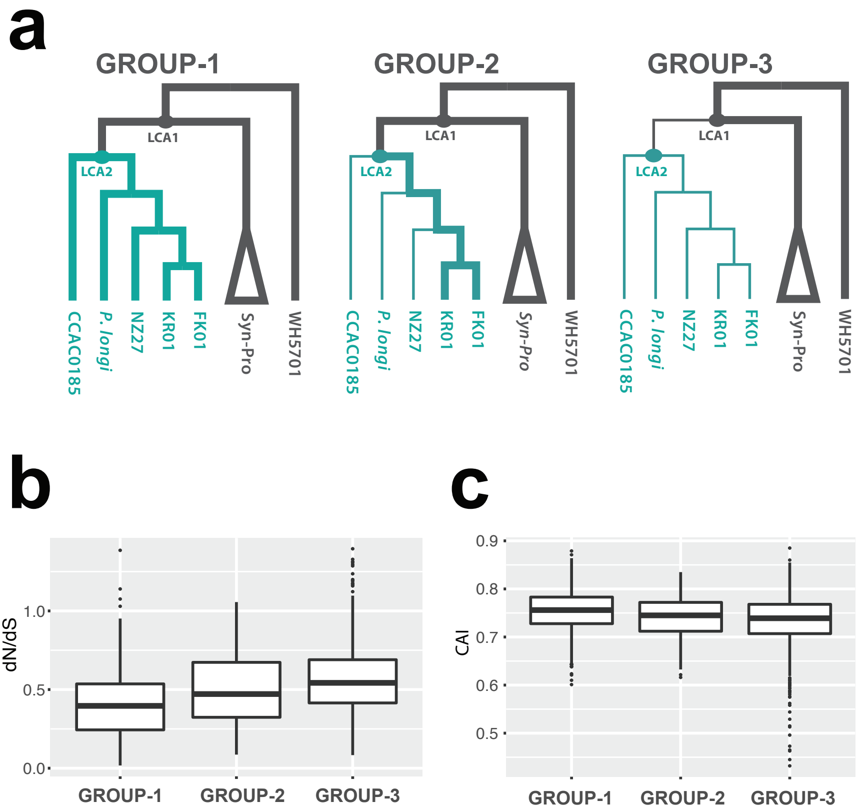

Figure S3. (a) Three scenarios for ancestral genes: (GROUP-1) gene present in all chromatophore genomes; (GROUP-2) gene present in LCA2 but lost in some of the photosynthetic *Paulinella* species – here is a particular example where a gene remains only in *P. micropora* KR01 and FK01; (GROUP-3) gene lost in all chromatophore genomes, presumably between LCA1 and LCA2. Thick lines represent presence and thin lines, absence of a gene. **(b)** Average ratio of substitution rates at non-synonymous and synonymous sites (dN/dS) calculated between free-living *Synechococcus* species (strain CC9616, WH 7805, and RS9916) for each loss scenario (GROUP-1, GROUP-2, GROUP-3). **(c)** Codon Adaptation Index (CAI) values of *Synechococcus* sp. WH5701 using codon usage table of *Synechococcus* sp. CC9311. CAI values were compared among three different categories; (GROUP-1, GROUP-2, GROUP-3)

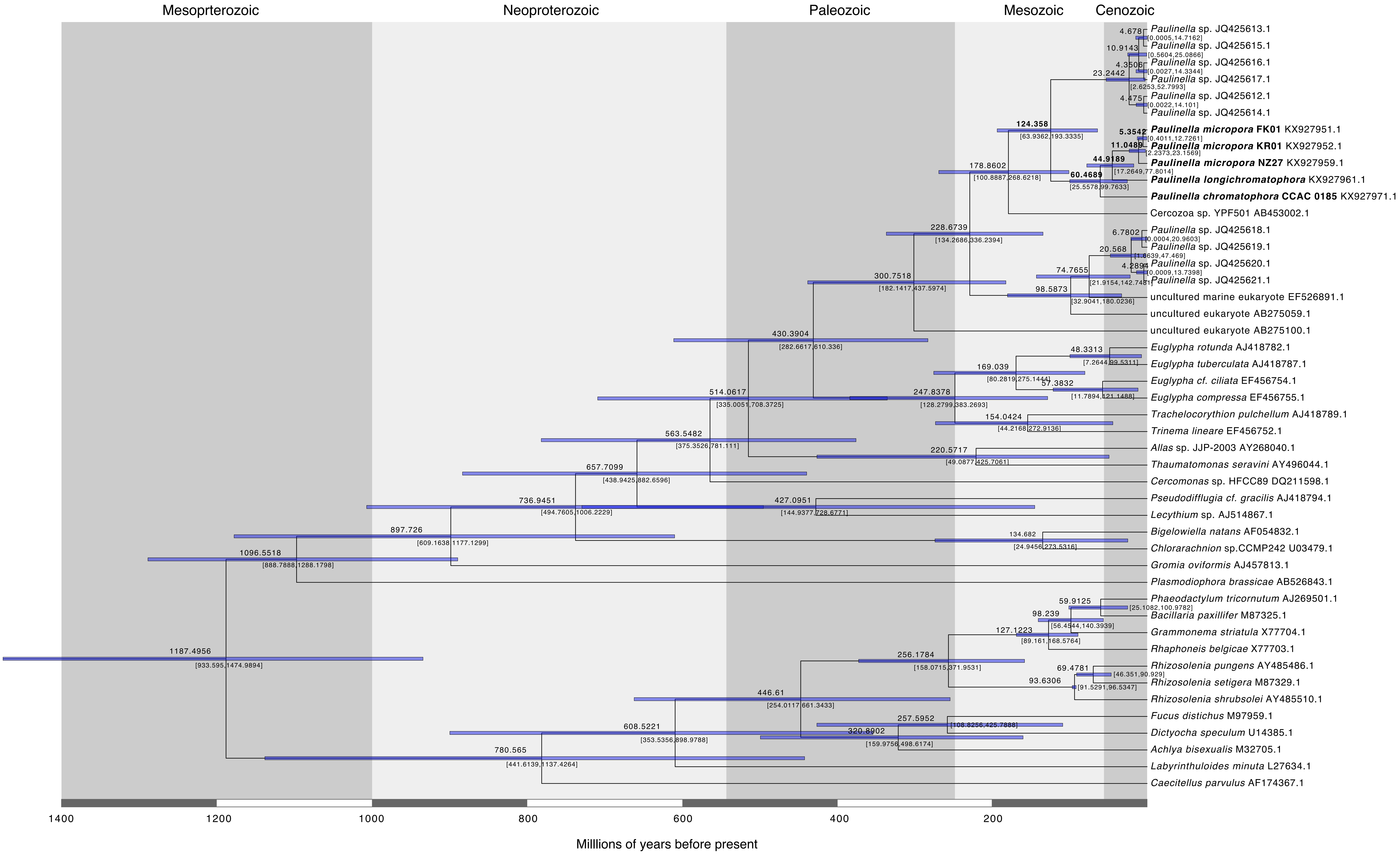

Figure S4. Divergence time estimation phylogram based on SSU rRNA sequence. Numbers at the nodes represent the mean estimated divergence time with the corresponding 95% highest posterior density of the nodes indicated by horizontal bars. The geological timescale is given in million years ago (Mya)

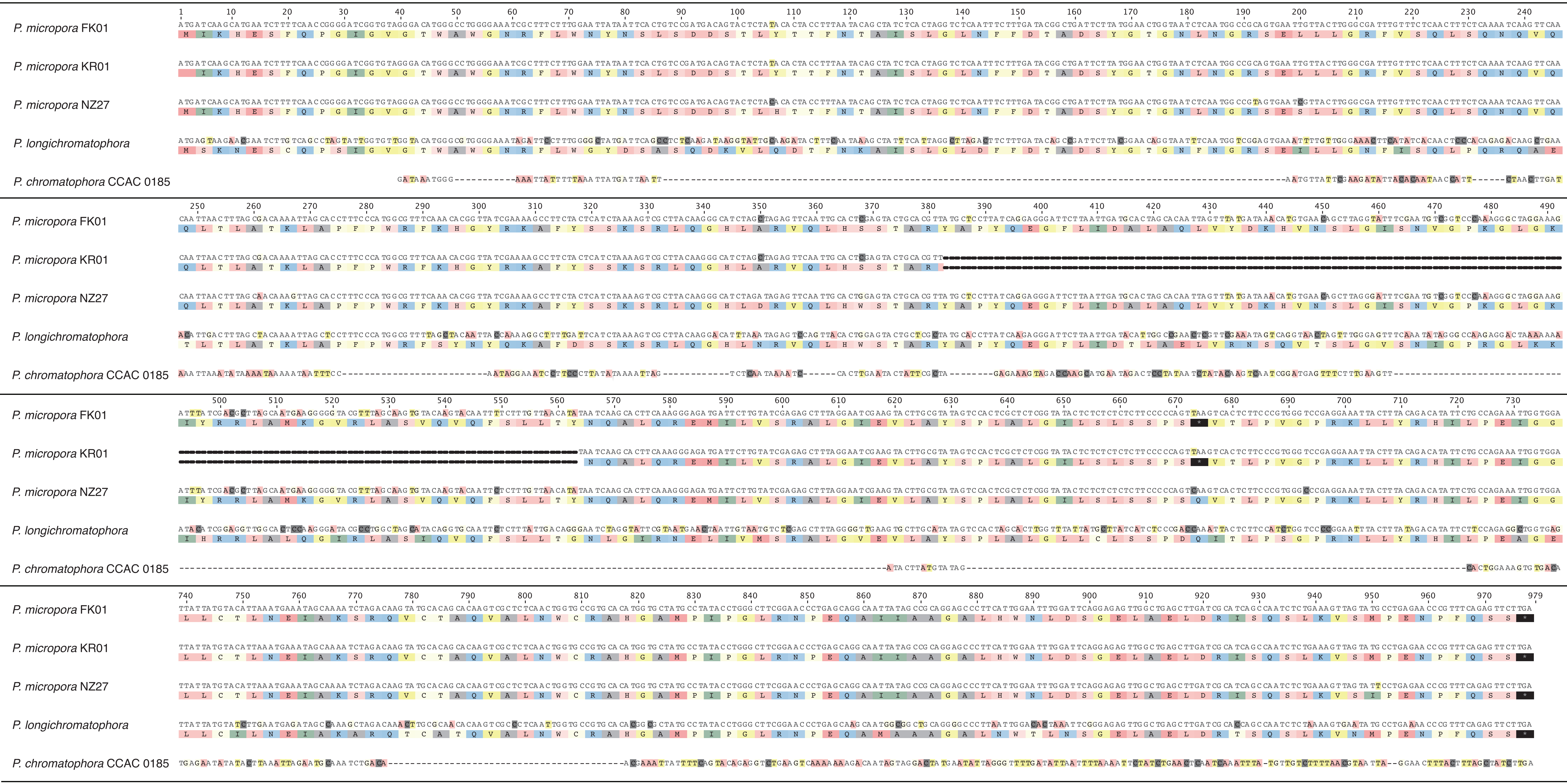

Figure S5. Oxidoreductase alignment of photosynthetic *Paulinella* lineages. Amino acids sequences are indicated below the nucleotide alignments. Black block is the stop codon. When there was difference among sequence alignment, we highlighted nucleotides that were different from the consensus sequence.

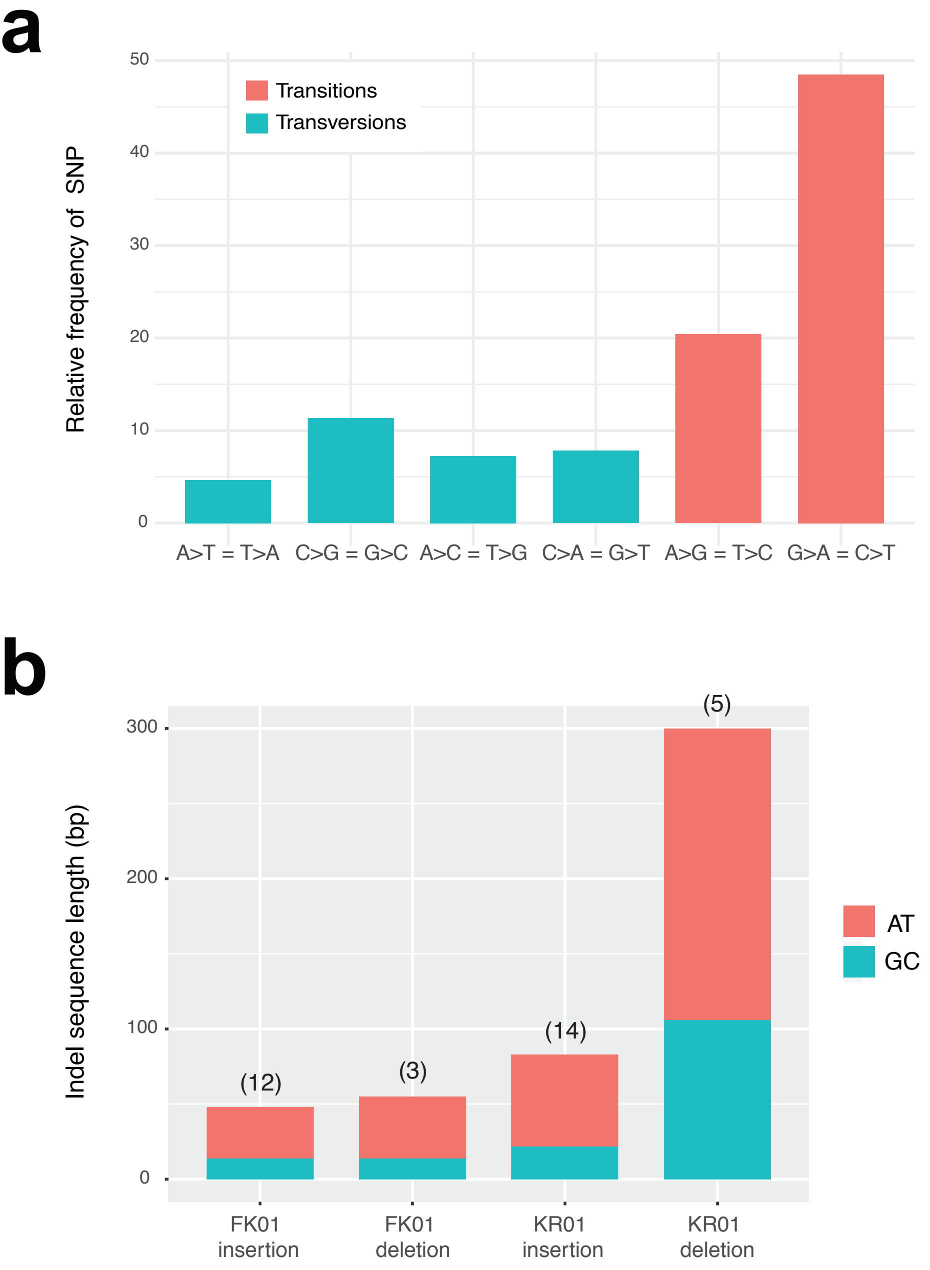

Figure S6. (a) Relative frequency of directional nucleotide substitutions in *Paulinella micropora* KR01 and *Paulinella micropora* FK01.
(b) Length of directional insertions and deletions in *Paulinella micropora* KR01 and *Paulinella micropora* FK01. Numbers inside the parentheses indicate the number insertion or deletion events.
